# THE EFFECTS OF AGE AND SIZE ON THE COMPANION DOG METABOLOME: ROLES OF TRYPTOPHAN AND FATTY ACID METABOLISM

**DOI:** 10.1093/geroni/igz038.1585

**Published:** 2019-11-08

**Authors:** Jessica M Hoffman, Steven Austad

**Affiliations:** Department of Biology, University of Alabama at Birmingham, Birmingham, Alabama, United States

## Abstract

Recently, the companion dog has been promoted as an ideal animal model for human aging. Dogs show an interesting phenomenon where smaller individuals are longer lived than their larger counterparts. However, many of the underlying molecular mechanisms that influence aging and longevity in the dog are unknown. To begin to uncover these physiological changes, we completed the largest metabolomics study to date in the companion dog. Here, we collected blood plasma samples from companion dogs in three in the United States for metabolomics analysis. We then looked at the effects of age and size on the metabolome to develop new hypotheses about healthy canine aging. Our most striking differences were found with regards to geographic location in the canine metabolome, in which metabolic profiles were more similar between dogs in the same city than across cities. After controlling for this location effect, we found a strong signal of amino acid metabolism, specifically tryptophan metabolism, associated with weight in the dog where metabolites in the tryptophan metabolism pathway were always higher in small, long-lived dogs. Future studies will directly investigate the role of tryptophan metabolism in model organisms.

